# Risk factors of suicidal spectrum behaviors in adults and adolescents with attention-deficit / hyperactivity disorder – a systematic review

**DOI:** 10.1186/s12888-023-05099-8

**Published:** 2023-08-21

**Authors:** Amalie Austgulen, Nanna Karen Gilberg Skram, Jan Haavik, Astri J. Lundervold

**Affiliations:** 1 Department of Biomedicine, Faculty of Medicine, University of Bergen, Jonas Lies Vei 91, 5009 Bergen, Norway; 2 Department of Biological and Medical Psychology, Faculty of Psychology, University of Bergen, Jonas Lies Vei 91, 5009 Bergen, Norway; 3 Division of Psychiatry, Haukeland University Hospital, Bergen, Norway

**Keywords:** Attention-deficit / hyperactivity disorder, ADHD, Suicide, Comorbidity, Adults, Adolescents, Self-harm, Suicidal spectrum behaviors

## Abstract

**Introduction:**

Adolescents and adults with attention-deficit/hyperactivity disorder (ADHD) are at increased risk of suicidal spectrum behaviors (SSBs). However, there is limited knowledge about risk factors triggering SSBs in this group of people.

**Objective:**

To explore published literature concerning factors that may increase the risk of SSBs in adults and adolescents with ADHD.

**Methods:**

A systematic literature search following Preferred Reporting Items for Systematic Reviews and Meta-Analyses (PRISMA) guidelines was conducted on 22^nd^ of February 2022 using the Ovid MEDLINE and Web of Science databases. Three categories of search terms were used: (1) self-harm, self-injury, self-mutilation, suicide, self-poisoning; (2) adults, adolescents; and (3) attention-deficit hyperactivity disorder/ADHD. Studies with data concerning mediating factors of SSBs in relation to a clinical diagnosis of ADHD in participants above 16 years of age were included.

**Results:**

The literature search identified 604 articles, of which 40 were included in the final study selection. Factors found to increase the likelihood of SSBs included ADHD symptom severity and persistence, female gender, family history of ADHD, childhood and parental influences, and social functioning. Even when adjusting for psychiatric comorbidities, most studies showed that adults and adolescents with ADHD have an elevated risk of SSBs.

**Conclusion:**

This systematic review has documented that several demographic and clinical features are associated with an increased risk of SSBs in adolescents and adults with ADHD. Notably, ADHD emerges as an independent risk factor for SSBs. This information ought to have clinical implications in terms of screening and suicide prevention strategies. Further longitudinal studies are needed to investigate the outcome of preventive strategies in individuals along the full spectrum of ADHD symptom severity.

**Supplementary Information:**

The online version contains supplementary material available at 10.1186/s12888-023-05099-8.

## Introduction

Suicide is a major cause of death, and suicidal spectrum behaviors (SSBs) are mental health problems causing harm at all societal levels [1]. Several recent studies and reviews have documented a strong association between having an attention-deficit/hyperactivity disorder (ADHD) and SSBs [1–6]. Identification of risk factors of SSBs in individuals with ADHD are thus essential, with clinical implications in terms of assessment procedures as well as treatment. This relationship inspired the present review to focus on factors associated with SSBs in adolescents and adults with ADHD.

ADHD is a common neurodevelopmental disorder, with an estimated global prevalence of 5.6% in school children and 2.6% in adults [7, 8]. Inattention and/or hyperactivity/ impulsivity are core symptoms of the disorder [9]. Having an ADHD diagnosis is also associated with challenges affecting social, academic and occupational success, high levels of stress and co-existence of other psychiatric disorders [2, 10]. Impairment of cognitive functions like decision-making and inhibitory control are also commonly reported [1, 2], and these problems are often embedded in a clinical presentation of emotional dysregulation [2, 10, 11]. Accordingly, many adolescents and adults with ADHD show challenges that are expected to play a role in triggering SSBs [2].

SSBs range from self-harm behaviors, suicidal thoughts and suicidal ideation (SI) to suicide attempts (SA) and completed suicides [12]. Self-harm behaviors, including non-suicidal self-injury (NSSI), self-injurious behaviors (SIB), and deliberate self-harm (DSH), are generally considered to be among the less severe SSBs. However, a systematic review concluded that self-harm predicts suicide [13], and that self-harm should be considered a major risk factor of suicide [14]. These findings call for increased awareness when self-harm is present, regardless of intent of behavior. Self-harm behaviors typically evolve during adolescence [2, 15, 16], while suicide is more prevalent towards late adolescence and adulthood [17]. The role of age, severity and persistence of ADHD symptoms, as well as other environmental and biological factors as potential predictors of SSBs are still unclear.

The risk of SSBs and increased morbidity have mainly been related to the high frequency of comorbid mood and anxiety disorders, substance use disorders and personality disorders among adults with ADHD [10, 14]. The results are, however, conflicting. While some studies find psychiatric comorbidities to play a mediating [5] or minor role [15], other studies point to comorbidities as important confounders [2]. A recent systematic review of longitudinal studies points to the uncertainty of the roles of comorbidities in ADHD as predictors of SSBs [4].

Previous reviews have mainly focused on the prevalence of SBBs [1–6, 13, 18], and have found a positive association between ADHD and various aspects of these behaviors. However, more information is needed regarding factors triggering SSBs to be better able to prevent adverse outcomes. To our knowledge, this is the first systematic review that investigates risk factors for SSBs in adolescents and adults with ADHD. By investigating a wide range of factors, such as psychiatric comorbidities, biological and environmental influences, gender and symptoms of ADHD, the present review takes on a broad approach to this task.

To that end, a systematic literature review following the Preferred Reporting Items for Systematic Reviews and Meta-Analyses (PRISMA) guidelines was conducted, focusing on factors contributing to increased risk of SSBs in adolescents and adults with ADHD. Ultimately, this information can be used to identify, prevent, and treat SSBs in individuals with ADHD.

## Methods

The primary literature search was conducted on the 22nd of February 2022, with the purpose of finding articles that examined the association between ADHD and SSBs in adults and adolescents. OVID Medline was chosen as the primary database and was accessed through the Norwegian Electronic Health Database.

### Search criteria

The following search criteria was entered: ("self harm" OR self-harm OR self-injur* OR "self injur*" OR suicid* OR self-mutilat* OR "self mutilat*" OR "self poison*" OR self-poison*) AND (adult* OR adolescen*) AND (ADHD OR "attention-deficit/hyperactivity" OR "attention-deficit hyperactivity" OR hyperkinetic).

### The screening process

**Fig. 1:**
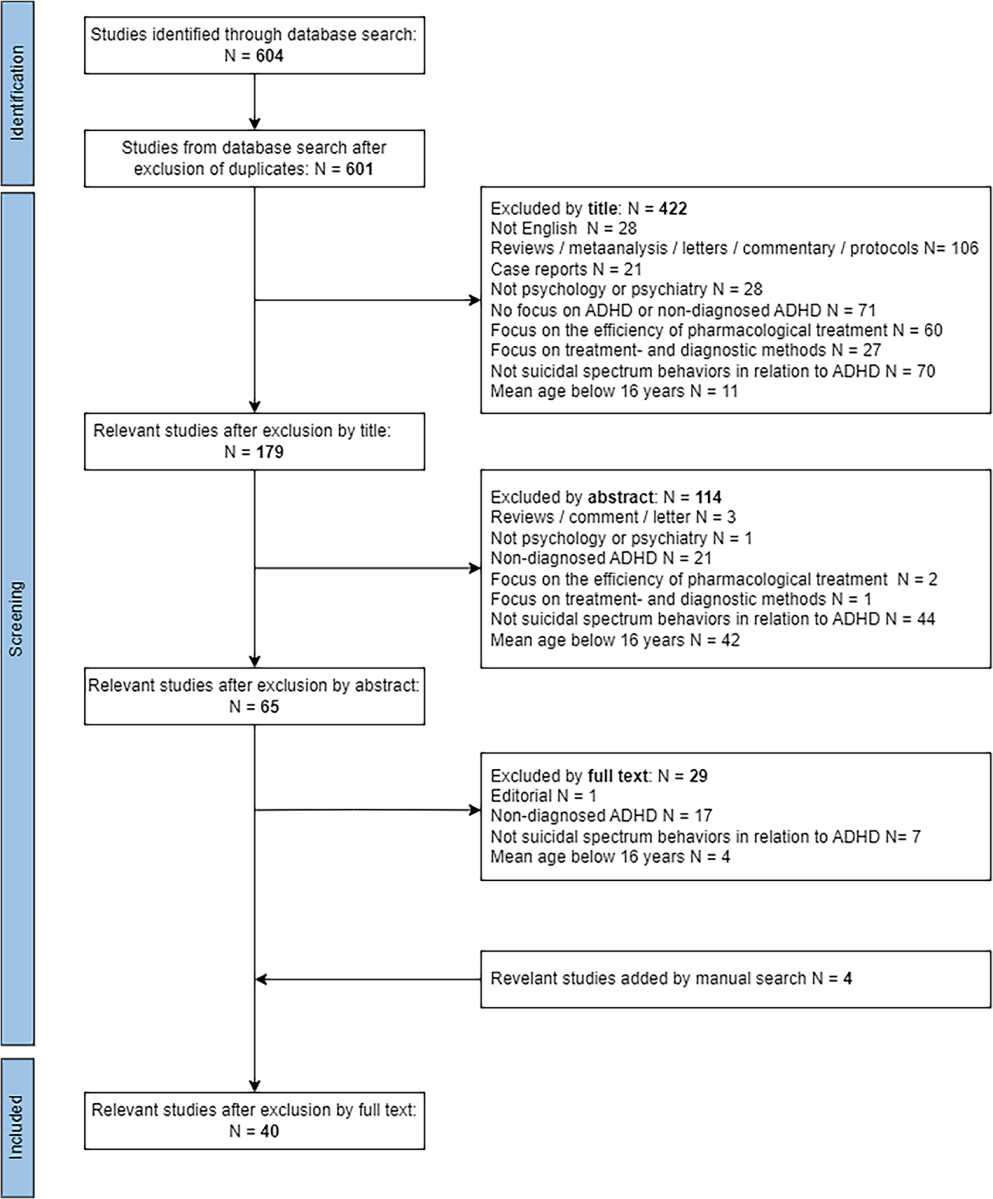
PRISMA Flowchart

OVID Medline returned a total of 604 articles (Fig. 1). In the initial screening, both first authors (AA and NKGS) screened the references for eligibility according to the inclusion and exclusion criteria. This was done independently in every step of the process. In cases where the authors disagreed respecting inclusion, the articles would be read and discussed again. If there was uncertainty or a continuous disagreement in terms of eligibility, AJL and JH were consulted for advice. All articles deemed relevant after exclusion were retrieved and independently studied for relevant results. Articles that were not available online were requested and retrieved.

An additional literature search was done the 13th of March 2022 on Web of Science, to examine any other relevant papers not included in the primary search. The same search criteria were used as in the previous literature search. This returned a total of 418 papers, and the references were screened by both authors. OVID Medline was repeatedly searched until the screening process was complete on the 27th of July 2022, to ensure that recently published papers could be included in the review.

In addition, all references from selected articles related to comorbid psychiatric disorders were searched to examine additional relevant results not included in the initial search. The same procedure was also done for all recent systematic reviews on the subject of ADHD and SSBs [1–6, 13].

The screening process is described in Fig. 1 and follows the PRISMA guidelines for systematic reviews.

### Inclusion criteria

- Papers written in English
- Study population with a mean age above 16, either at baseline or at assessment for SSBs
- Inclusion of an ADHD sample with a diagnosis according to DSM (DSM-III, III-R, IV, IV-TR, 5) or ICD (-8, -9 or -10) criteria
- Data on factors associated with ADHD that mediate the risk of SSBs

### Exclusion criteria

- Reviews, meta-analyses, comments, letters, editorials, case-reports, and protocols
- Not in the field of psychology or psychiatry
- Studies investigating treatment- and diagnostic methods
- Studies investigating efficiency of pharmacological treatment

A clinical diagnosis of ADHD was required to ensure validity. Articles only reporting ADHD symptoms or diagnoses solely based on self-report questionnaires, such as the Adult ADHD Self-Report Scale (ASRS) or Wender Utah Rating Scale (WURS), were thus excluded. We included studies with a clinical ADHD diagnosis collected from registers or medical prescriptions indicating ADHD. Studies comparing comorbid psychiatric disorders with and without ADHD were included (e.g., only depression vs. depression + ADHD), while studies without a clear differentiation between diagnoses were excluded.

## Results

The literature search returned a total of 604 papers, including three duplicates (Fig. 1). One paper was added manually after screening systematic reviews on the same topic [19], while two papers were added because they were published after the initial search [20, 21]. From the search on Web of Science, one paper was found to be relevant and added to the final study selection [22]. In total, 40 studies were included in the systematic review after the screening process was completed. An overview of the methods and main findings for each study can be found in Supplementary Table 1.

### Study characteristics

The majority of the studies (23/40) were published between 2017 and 2022 [20–42]. The studies reported data from 14 different countries across five continents. The United States and Sweden were the most common countries of origin, with thirteen and seven published papers included in this review, respectively [20, 23, 24, 29, 30, 33, 34, 37, 39, 40, 42–51].

In total, the papers investigated 15 million individuals, of which there were 626 663 individuals diagnosed with ADHD. This number was reached after excluding papers that most likely used the same study populations. Thirty-seven articles included information about number of participants, of which 52% were males. Thirty-four articles also reported the number of included participants with ADHD, with an estimated percentage of males at 62%.

### Assessment of ADHD

Different instruments were used for assessing and establishing an ADHD diagnosis, including clinical interviews, national registers, or databases. All methods followed the ICD- or DSM-diagnostic criteria. See Table 1 for further details about the assessment methods and tools included.

**Table 1:** Overview of the methods used to assess for a diagnosis of ADHD

Method	Number of studies
*Clinical assessment or interview conducted by clinicians*	
According to DSM-IV or DSM-IV-TR criteria	8
Extensive clinical process in childhood	7
Total:	**15**
*Identified through registers or databases*	
ICD-codes in national registers (Sweden and Denmark)	7
ICD-codes in Taiwan National Health Insurance Research Database	1
MarketScan Commerical Claims and Encounters database	1
TIC database	1
Total:	**10**
*Use of instruments to aid in establishing an ADHD diagnosis*	
Conner's Adult ADHD Diagnostic Interview for DSM-IV (CAADID)	3
Kiddie—Schedule for Affective Disorders and Schizophrenia—present-life -version (K-SADS-PL)	2
Structured Clinical Interview for DSM (SCID-I and SCID-II)	3
Mini-International Neuropsychiatric Interview (MINI and MINI-Plus)	2
Diagnostic Interview for ADHD in Adults 2.0 (DIVA 2.0)	1
Semi-structured Assessment for Drug Dependence and Alcoholism (SSADDA)	1
Total:	**12**
*Other methods*	
Self-report—if participants had been diagnosed by a health professional	1
Medical records	1
Recorded history of symptoms, clinical diagnosis, results on questionnaires and documentation of medical treatment	1
Total:	**3**

### Definitions of SSBs

Since SSBs are a broad concept, several studies have investigated more than one aspect of SSBs. This accounts for 14 of the studies included in the review [23, 26, 28, 29, 34, 35, 37–39, 43–46, 49].

A wide range of definitions of self-injurious or suicidal behaviors are used in the final selection of studies. The majority, which accounts for 35 of 40 articles, focused on suicide as one of their outcomes. SA or acts are the most common behavior investigated. We identified 12 studies that investigated SI or thoughts as one of their outcomes [23, 26, 28, 31, 32, 34, 35, 38, 39, 41, 43, 45], while 11 studies focused on aspects of self-injurious behavior [28, 29, 33, 34, 37, 43–46, 49, 52] (Table 2).

**Table 2:** Overview of the different aspects of suicidal spectrum behaviors (SSBs) investigated

Aspect of SSBs	Number of studies
*Suicidal ideation*	
- Suicidal ideation (SI)	11
- History of suicidal thoughts / behavior	1
Total:	**12**
*Self-harm behaviors*	
- Non-suicidal self-injury (NSSI)	7
- Self-injury/intentional self-injury	2
- Self-injurious behavior (SIB)	1
- Deliberate self-harm (DSH)	1
Total:	**11**
*Suicide*	
- Suicide attempts (SA) or suicidal acts	26
- Completed suicide (mainly as cause of death)	6
- Suicidality/suicide risk	5
- Suicidal behavior	1
- Familial psychiatric history of suicide	1
Total:	**35**

### Prevalence of SSBs in ADHD

Of the included studies, thirteen investigated the prevalence and impact of an ADHD diagnosis on SSBs among adolescents and adults [20, 22–24, 27–29, 33, 34, 40, 44, 48, 50].

#### Suicidal ideation

ADHD was found to be associated with a higher likelihood of SI, with odds ratios (ORs) ranging from 1.83 (95%CI [1.69–1.97]) to 8.48 (95%CI [1.00–74.07]) [23, 39]. Participants with an ADHD diagnosis also reported more frequent SI than controls (46.0% vs. 5.9%, *p* < 0.001) [28].

In a study conducted by Meza et al*.* [34], participants with ADHD were not found to have significantly higher SI lifetime risk than controls (OR 1.4, 95% [0.82–2.51]).

#### Self-harm behaviors

Older adolescents and adults with ADHD had a higher rate of self-injurious behavior without suicidal intent (*p* = 0.033) and a higher NSSI lifetime risk (OR 3.1, 95% CI [1.74–6.15]), when compared to control groups [28, 34]. A significant relationship between ADHD status in childhood and NSSI frequency (*p* < 0.001) and NSSI severity (*p* < 0.001) emerged when controlling for age, maternal education, and family income [29]. Laporte et al*.* found that having ADHD in adulthood was significantly associated with DSH in a sample of young violent offenders (*p* = 0.005), but this result did not remain statistically significant after multivariate adjustments [33]. When investigating adults presenting with self-harm in Swedish hospitals, NSSI was found to be more common among adults with ADHD than those without [20].

#### Suicide

Ten studies showed a significantly increased likelihood of SA among individuals with ADHD [20, 22–24, 27, 28, 34, 39, 40, 50]. In a Swedish population study, persons with ADHD had an increased risk of both attempted suicide (OR 8.46, 95% CI [8.07–8.87]) and completed suicide (OR 12.22, 95% CI [8.67–17.22]) when compared to controls [50]. This result remained significant after adjusting for psychiatric comorbidity [50]. Similarly, Sun et al*.* found that having an ADHD diagnosis was associated with an 8.63-fold (95% CI [6.27–11.88]) increased risk of death by suicide [40]. In a study from Denmark, individuals with ADHD were estimated to have a 4.7-fold higher rate of suicidal behavior (95%CI [4.34–5.06]) and an increased risk of death by suicide (IRR 3.20, 95% CI [2.29–4.47]) [22]. Other studies similarly reported significantly higher rates of SAsuicide attempts in those with an ADHD diagnosis compared to those without, with unadjusted odds ratios ranging from 2.27 to 13.50 [23, 27, 28, 34, 39].

When investigating adults presenting with self-harm at Swedish hospitals, Olsson et al. reported that 29% of those with ADHD had at least one SA or death recorded six months after the initial self-harm episode, which was a significantly higher proportion than in the comparison group (19%, *χ2* = 4.67, *df* = 1, *p* = 0.03) [20]. In the final regression model, the presence of ADHD was not found to be associated with suicidal behavior during follow-up after adjusting for a clinical diagnosis of depression and emotionally unstable personality disorder (EUPD) at the baseline (OR 1.58, 95% CI [0.96–2.60], *p* = 0.073) [20].

### ADHD related characteristics

#### ADHD presentations

Six studies explored ADHD subtypes in relation to SSBs [34, 35, 44, 47, 49].

##### Suicidal ideation

Oh et al*.* compared the prevalence of SI in individuals with predominantly inattentive presentation (ADHD-I) and those with a combined inattentive and hyperactive/impulsive presentation (ADHD-C) [35]. They showed that level of emotional symptoms in participants with ADHD-C was associated with an increased frequency of recurrent thoughts and SI (adjusted OR 22.57, 95%CI [5.04–100.99], *p* < 0.001) [35]. In the Berkeley Girls with ADHD Longitudinal Study (BGALS) sample, girls with ADHD-C presentation showed higher rates of lifetime SI when compared to the ADHD-I subgroup (OR 3.1, 95%CI [1.38–6.77], *p* = 0.004) [34].

##### Self-harm behaviors

Regarding self-injury, adolescents and adults with ADHD-C presentation were found to have increased likelihood of NSSI, both regarding frequency (*p* < 0.001) and severity (*p* < 0.001) [49], as well as a higher lifetime risk of NSSI (OR 2.4, 95%CI [1.11–5.09]) than controls [34].

##### Suicide

Adolescents and adults with ADHD-C presentation had a higher rate of SA (15–22%) when compared to individuals with ADHD-I presentation and those without ADHD [44, 47].

#### Persistence of ADHD symptoms

Persistent symptoms from childhood into adolescence was studied in two articles including 228 females in the BGALS sample [37, 49]. Girls with persistent ADHD showed significantly higher frequency, variety and severity of NSSI than girls with transient ADHD and the girls in the comparison group (*p* < 0.001), and they had higher rates of SA than the comparison group (*p* < 0.01) [49]. Owens et al. followed up by showing that females with diagnostic persistence from childhood to adulthood had significantly higher risks of both NSSI and SA when compared to those without ADHD (OR 6.0 and 5.8) and those with an age-related decline in ADHD symptoms (OR 6.1 and OR 10.6) [37].

#### ADHD symptoms

Two studies investigated the role of inattention and/or impulsivity in relation to self-harm and attempted suicide in individuals with ADHD [20, 34]. Inattention and hyperactivity/impulsivity score on the Swanson, Nolan, and Pelham Questionnaire (SNAP), measured in childhood, were found to be significantly correlated with lifetime NSSI (*p* < 0.001) and SA (*p* < 0.05) [34]. Olsson et al. found that the impulsivity trait, measured with the Suicide Assessment Scale (SUAS-11), was significantly more common among adults presenting with both self-harm and ADHD, when compared to those without ADHD. This was also the case for impulsivity in connection with the initial self-harm episode in those with SA (*p* = 0.01). The trait impulsivity did not remain a significant predictor in regression models (OR 1.15, 95% CI [0.79–1.68], *p* = 0.47) when adjusting for a clinical diagnosis of depression and emotionally unstable personality disorder (EUPD) at baseline, sex and age [20].

#### Executive functioning

Three studies showed that aspects of executive functioning (EF) were significantly associated with the presence of SSBs in individuals with ADHD [34, 45, 46]. Meza et al*.* investigated the impact of response inhibition (RI) in two of their studies [34, 45]. In their article from 2016, RI was found to be positively associated with SI, SA and NSSI (*p* < 0.05), and to act as a significant predictor of SA and NSSI severity in late adolescence and early adulthood [45]. In an article from 2021, Meza et al*.* reported that a global measure of EF was significantly correlated with lifetime NSSI (*p* = 0.012) [34]. Miller et al*.* further supported an association between EF and NSSI/SA (*p* = 0.016 and *p* = 0.043, respectively) in a study including a wider range of psychometric tests of EF [46].

#### Internalizing and externalizing symptoms

Three studies investigated, among other factors, internalizing and externalizing symptoms in females with and without ADHD [29, 34, 49]. The Child Behavior Checklist (CBCL) was distributed during childhood and adolescence and compared with emerging results during adolescence and early adulthood [29, 34, 49].

Swanson et al*.* showed that externalizing symptoms were found to partially mediate the relation between ADHD status in childhood and NSSI severity in late adolescence and early adulthood. Internalizing symptoms emerged as a significant partial mediator between ADHD status and SA [49]. In 2017, Gordon and Hinshaw found that the association between ADHD status and NSSI frequency and severity remained significant when accounting for externalizing symptoms [29]. Similarly, Meza et al*.* revealed that the CBCL externalizing scores were a significant predictor of NSSI, qualified by internalizing scores and measures of EF. Among those with high CBCL externalizing and internalizing scores and poor EF, 80% were found to have a lifetime history of NSSI [34].

### Gender differences

Gender differences in relation to ADHD and SSBs were investigated in six studies, all reporting that females with ADHD have a higher likelihood of SSBs than males with ADHD [22, 23, 27, 32, 36, 50].

#### Suicidal ideation

Kakuszi et al*.* investigated the role of gender differences in the association between ADHD and SI in 206 participants [32]. Females with ADHD were found to have a significantly higher likelihood of SI than males with ADHD (OR 25.0 vs OR 2.90). This remained significant after adjusting for age, comorbidities, and treatment with methylphenidate. “Problems with self-concept” scores on Conners’ Adult ADHD Rating Scales (CAARS) were most closely associated with SI in females (OR 5.60, 95% CI [2.34–13.41]), while the SI association was related to “impulsivity” scores in males (OR 3.01, 95% CI [1.50–6.06]) [32].

In a study conducted by Babinski et al*.*, a significant ADHD * sex interaction emerged for SI (*p* < 0.0001). ADHD was associated with SI among females (OR 2.21, 95% CI [1.95–2.49]), with a higher odds ratio than in males (OR 1.61, 95% CI [1.46–1.77]) [23].

#### Suicide

In the same study, female gender was significantly associated with a higher likelihood of SA (OR 1.52, 95% CI [1.22–1.90]) [23]. A study from 2020 reported similar results, with female gender being one of the significant positive correlates of SA among adults with ADHD [27].

A Danish population study found that females with ADHD had a 9.06-fold higher rate of suicidal behavior (95% CI [8.12–10.12], *p* < 0.001) than males without ADHD. Males with ADHD had 3.38-fold higher rate than those without ADHD (95% CI [3.04–3.76], *p* < 0.001) [22]. In another study from Denmark, suicidal behavior was found to be more common in females with ADHD than males with ADHD (IR 124.38, 95% CI [113.17–136.69] vs. IR 41.03, 95% CI [37.67–44.69]). Additionally, the association with ADHD was significantly stronger in females than in males, with a hazard ratio of 1.28 (95% CI [1.12–1.47], *p* < 0.001) [36]. In a Swedish population study, the risk of attempted suicide in ADHD participants were found to differ significantly by gender (*p* < 0.001), with the highest risk in females (OR 5.41, 95% CI [4.60–6.36]) [50].

#### Psychiatric comorbidity

#### Risk of SSBs in individuals with ADHD and comorbid psychiatric disorders

##### Suicidal ideation

Kakuszi et al*.* found no significant differences between those with and without comorbid psychiatric disorders regarding the risk of SI [32] (Table 3). However, the presence of depression, bipolar or anxiety disorders was associated with a numerically higher ORs for SI in females [32] (Table 4).

**Table 3:** Overview of risk estimates when including or adjusting for the presence of psychiatric comorbidities in adolescents and adults with ADHD

	**Studies**	**SSBs**	**Baseline risk estimates [95%CI]**		**Psychiatric comorbidities [95%CI]**	**Comment**
*Inclusion of psychiatric comorbidities*	Fitzgerald C. et al*.* (2019) [22]	*Suicidal behavior*	IRR 4.09 [3.53–4.73]	**↑**	IRR **10.43** [9.53–11.41]	Reference: No psychiatric disorder (IRR 1.0)
	Kakuszi B. et al*.* (2018) [32]	*Suicidal ideation*	ADHD only OR 1.0 (reference)	***NS***	Males: OR 1.11 [0.25–4.85] Females: OR 2.64 [0.64–10.87]	
	Sun S. et al*.* (2019) [40]	*Suicide*	ADHD only HR 1.0 (reference)	**↑**	HR **9.10** [3.95–20.98] * HR **8.96** [3.89–20.66] **	*Adjusted for birth year and sex **Further adjusted for birth weight, maternal age at birth, parental educational level, and parental employment status
*Adjustment for psychiatric comorbidities*	Fuller-Thomson E. et al*.* (2020) [27]	*Suicide attempt*	OR 3.27 [2.39–4.48] *	**↓**	OR **1.56** [1.08–2.25] **	*Only adjusted for demographics and socioeconomic status **Further adjusted for lifetime history of mental illness, chronic pain, and childhood adversities
	Hirvikoski T. et al*.* (2020) [30]	*Suicide attempt*	ASD + ADHD: OR 7.25 [6.79–7.73] ASD + ID + ADHD: OR 5.60 [4.70–6.68]	**↓**	ASD + ADHD: OR **2.31** [2.11–2.53] ASD + ID + ADHD: OR **2.90** [2.36–3.57]	Adjusted for depression, anxiety, and SUD
	Ljung T. et al*.* (2014) [50]	*SA and CS*	SA: OR 8.46 [8.07–8.87] CS: OR 12.22 [8.67–17.22]	**↓**	SA: OR **3.62** [3.29–3.98] CS: OR **5.91** [2.45–14.27]	Adjusted for comorbid psychiatric disorders
	Olsson P. et al*.* (2022) [20]	*Suicide attempt*	OR 1.70 [1.05–2.79]	***NS***	OR 1.58 [0.96–2.60]	Adjusted for depression and EUPD
	Ruchkin V. et al*.* (2017) [39]	*SI and SA*	SI: OR 8.84 [1.00–74.07] SA: OR 13.50 [1.53–119.02]	**↓**	SI: OR 0.18–10.61 * SA: OR 0.342–17.67 **	*Significant: ADHD x drug dependence (OR 10.61, 95%CI [1.39–80.73]) **Significant: ADHD x alcohol dependence (OR 9.61, 95%CI [1.58–58.27])
	Yoshimasu K. et al*.* (2019) [42]	*Suicidality*	OR 2.42 [1.51–3.86]	**↓**	OR **1.94** [1.19–3.15]	Adjusted for the presence of any of the psychiatric disorders included in the study

*SSBs* Suicidal spectrum behaviors, *SA* Suicide attempt, *CS* Completed suicide, *SI* Suicidal ideation, *NS* Non-significant, *HR* Hazard Ratio, *OR* Odds Ratio, *IRR* Incidence Rate Ratio, *SUD* Substance use disorder, *EUPD* Emotionally unstable personality disorder

**Table 4:** Overview of risk estimates when including or adjusting for different psychiatric comorbidities in adolescents and adults with ADHD

	**Study**	**SSBs**	**Baseline risk estimates [95%CI]**		**SUD** **[95%CI]**	**Depression** **[95%CI]**	**Anxiety disorders [95%CI]**	**Bipolar disorder [95%CI]**	**Personality disorder [95%CI]**	**Autism spectrum disorders** **[95% CI]**	**Schizophrenia disorders [95%CI]**
*Inclusion of specific psychiatric comorbidities*	Fitzgerald et al*.* (2019) [22]	*Suicidal behavior*	ADHD without specified comorbidity IRR 2.63–2.90 [2.27–3.36]	**↑**	IRR **21.55** [17.98–25.83]	IRR **13.85** [8.48–22.61]	*Anxiety:* IRR **7.02** [6.16–7.98] *OCD:* IRR **5.35** [2.78–10.28] *PTSD:* IRR **27.79** [15.78–48.95]	IRR **13.85** [8.48- 22.61]	IRR **17.25** [14.32–20.77]	IRR **4.65** [3.25–6.66]	IRR **17.26** [12.27–24.29]
	Fuller-Thomson et al*.* (2020) [27]	*Suicide attempt*	ADHD without specified comorbidity OR 1.0 (reference)	**↑/*NS***	OR **2.35** [1.23–4.49]	OR **7.06** [3.52–14.16]	OR 0.94 [0.46–1.92]				
	Kakuszi et al*.* (2018) [32]	*Suicidal ideation*	ADHD only OR 1.0 (reference)	***NS***		Males: OR 0.87 [0.17- 4.72] Females: OR 3.64 [0.75–17.9] *("Affective disorders")*					
	Sun et al. (2019) [40]	*Suicide*	ADHD only HR 1.0 (reference)	**↑ /*NS***	HR **6.65** [4.16–10.62]	HR **4.22** [2.71–6.55]	HR **5.84** [3.71–9.17]	HR **6.18** [3.55–10.77]	HR **6.69** [4.03–11.10]	HR 1.13 [0.63–2.00]	HR **3.59** [1.88–6.85]
*Adjustment for specific psychiatric comorbidities*	Ruchkin et al. (2017) [39]	*SI and SA*	SI: OR 8.84 [1.00–74.07] SA: OR 13.50 [1.53–119.02]	**↓/↑/*NS***	*Alcohol* SI: OR 1.33 [0.21–8.53], SA: OR **9.61** [1.58–58.27] *Drug* SI: OR **10.61** [1.39–80.73], SA: OR 0.36 [0.05–2.71]	SI: OR 4.46 [0.54–36.55] SA: OR 0.34 [0.37–3.14]	*Anxiety* SI: OR 0.80 [0.09–6.12], SA: OR 0.73 [0.07–7.62] *PTSD* SI: OR 0.09 [0.01–1.78], SA: OR 0.78 [0.09–6.45]	SI: OR 0.18 [0.02–2.27] SA: OR 17.67 [0.91–341.60] *("Mania")*			
	Yoshimasu et al*.* (2019) [42]	*Suicidality*	OR 2.42 [1.51–3.86]	**↓/↑**	*Substanse-related* OR **2.16** [1.34–3.48] *Alcohol dependence* OR **2.37** [1.48–3.79]	OR **1.93** [1.18–33.15]	*GAD*: OR **2.27** [1.40–3.67] *OCD:* OR **2.12** [1.31–3.44] *PTSD:* OR **2.28** [1.41–3.69]	*Hypomanic episode:* OR **2.06** [1.31–3.78], *Dysthymia:* OR **2.06** [1.27–3.35]	*APD:* OR **2.20** [1.36–3.55]		

*SSBs* Suicidal spectrum behaviors, *SA* Suicide attempt, *SI* Suicidal ideation, *NS* Non-significant, *HR* Hazard ratio, *OR* Odds ratio, *IRR* Incidence rate ratio, *SUD* Substance use disorder, *OCD* Obsessive compulsive disorder, *PTSD* Post-traumatic stress disorder, *GAD* General anxiety disorder, *APD* Antisocial personality disorder

In a study of juvenile delinquents with and without ADHD, comorbid drug dependence was associated with a higher likelihood of SI [39].

##### Self-harm behaviors

A study including 804 adults who presented with self-harm at three Swedish hospitals were investigated at the initial self-harm episode and at six-month follow-up [20]. Compared to adults without an ADHD diagnosis, adults with ADHD were less likely to have a clinical diagnosis of depression at discharge but had significantly higher mean scores on a depression scale (MADRS-S) and higher rates of personality disorders. A binary logistic regression model revealed a 70% increase in odds for suicidal behavior among participants with ADHD (OR 1.70, 95%CI [1.05–2.76], x^2^ = 4.59, *p* = 0.03). The OR remained elevated after adjustment for clinical diagnosis of depression at baseline (OR 1.65, 95%CI [1.01–2.68], x^2^ = 3.97, *p* = 0.046), but was no longer significant after adjustment for EUPD (OR 1.58, 95%CI [0.96–2.60], x^2^ = 3.22, *p* = 0.073) [20].

##### Suicide

Eight of the included studies investigated a wide range of psychiatric comorbidities in relation to ADHD and suicide [20, 22, 27, 30, 39, 40, 42, 50]. Two of the studies compared the risk of SSB in individuals with ADHD with the risk in those with both ADHD and comorbid psychiatric disorders [22, 40].

A large Swedish prospective cohort study including 86 670 individuals with ADHD born between 1983 and 2009 found that the association between ADHD and all-cause mortality increased substantially with number of psychiatric comorbidities [40]. Similarly, a Danish population study reported an increased rate of suicidal behavior in individuals diagnosed with additional psychiatric disorders [22]. In six studies adjusting for the presence of psychiatric comorbidities [20, 27, 30, 39, 42, 50], the risk estimates decreased, but remained significant in four studies [27, 30, 42, 50] (Table 3).

Six studies analyzed effects of multiple comorbidities (Table 4). The Canadian Community Health Survey found that having a substance use disorder (SUD) or a lifetime history of depression was positively correlated with suicide among adults, but this was not the case for anxiety [27]. Having comorbid SUD also yielded the highest rate of suicidal behavior in a Danish population study and in a Swedish register-based study [22, 40]. In a study conducted by Ruchkin et al*.*, comorbid alcohol dependence increased the likelihood of SA in older adolescents with ADHD [39].

Conflicting findings were reported for the role of anxiety and depression. In two of the included studies, adjustment for comorbid anxiety was associated with a lower or non-significant likelihood of SA [27, 39]. In contrast, the presence of comorbid anxiety was associated with an increased likelihood of suicidal behavior or suicide in three studies [22, 40, 42]. Four studies found a significant effect of depression on the likelihood of suicide [22, 27, 40, 42], while one study did not [39] (Table 4).

##### Risk of SSBs in individuals with psychiatric disorders and comorbid ADHD

A total of 16 articles investigated risk of SSBs in individuals with ADHD as a comorbid disorder to another psychiatric disorder, such as a major depressive disorder (MDD), bipolar disorder (BD), anxiety disorders, SUD, Tourette syndrome (TS) and chronic fatigue syndrome (CFS) [19, 25, 26, 31, 38, 41, 43, 51–58]. One study compares individuals who have ADHD with and without comorbid anxiety disorders [21].

###### Suicidal ideation

In a study conducted by Delibas et al*.* [26]*,* patients with MDD and ADHD had significantly higher rates of SI (*p* < 0.018)) than patients without comorbid ADHD. There were no significant differences between adults with and without comorbid ADHD in a clinical sample of bipolar patients (*p* = 0.24) [38].

Three articles focused on the association between SUD, comorbid ADHD, and SI [31, 41, 43]. Arias et al*.* investigated 1 761 adults with a lifetime diagnosis of cocaine and/or opioid dependence and found that a comorbid ADHD diagnosis was significantly associated with SI (*p* < 0.001) [43]. The other studies found no significant differences between the two patient groups [31, 41]. Interestingly, Icick et al*.* found that having ADHD in adulthood was significantly associated with increased severity profiles of SUDs, borderline personality disorder (BPD) and a number of other comorbid disorders [31]. Similarly, Umar et al*.* found that the combined group had poorer quality of life, more history of aggression and an increased number of drug relapses [41].

###### Self-harm behaviors

In the study conducted by Arias et al*.,* a comorbid ADHD diagnosis was significantly associated with intentional self-injurious behavior [43]. When investigating TS, adults with comorbid ADHD were more likely to engage in SIB (*p* = 0.001) [52].

###### Suicide

Patients with MDD and ADHD were more likely to have engaged in SA (*p* < 0.001) than patients without comorbid ADHD, and showed higher mean scores on Hamilton Depression Rating Scale (HDRS) during admission and discharge [26]. Harmanci et al*.* examined patients with BD or MDD and compared SA in the participants with and without comorbid ADHD [53]. The impact of comorbid ADHD diagnosis was significant in patients with BD (*p* = 0.039) but not MDD (*p* = 0.051) [53]. McIntyre et al*.* investigated a similar group of MDD and BD patients, which showed no significant differences in terms of suicide risk in patients presenting with or without comorbid ADHD [19].

Six additional studies [38, 51, 54–57] compared patients with BD with and without comorbid ADHD. While two studies showed non-significant differences regarding suicidal behavior [51, 57], four studies revealed a significantly higher risk of SSBs in those with comorbid ADHD [38, 54–56]. In a study by Pinna et al*.* [38], adults with ADHD and BD had a significantly higher risk of suicidal acts (48%, *p* = 0.008). Torres et al*.* confirmed that the combined group was more likely to have attempted suicide (*p* = 0.030) and to have a history of suicidal behavior (*p* = 0.006), but this group also showed a lower proportion of severe SA compared to the BD-only group (15.4% vs. 46.7%, *p* = 0.52) [56]. Lan et al*.* found that the presence of ADHD emerged as an independent risk factor for attempted suicide in adolescents and young adults with BD after adjusting for demographic factors and other psychiatric comorbidities [55].

Quenneville et al*.* investigated 353 adults with ADHD. Those with comorbid anxiety disorders were significantly more likely to have a history of SA (OR 2.49, 95% CI [1.79–3.43], *p* < 0.001). After adjustment for age, gender and other comorbid disorders, including BD, BPD and MDD, they found that post-traumatic stress disorder (PTSD) had the strongest association with a history of SA (OR 3.55, 95% CI [1.93–6.51], *p* < 0.001), followed by panic disorders (OR 2.72, 95% CI [1.58–4.69], *p* < 0.001) and social phobia (OR 2.51, 95% CI [1.47–4.27], *p* = 0.001) [21].

Blanco-Vieria et al. studied the impact of comorbid ADHD in patients with obsessive–compulsive disorder (OCD). Their findings showed that a combined OCD and ADHD led to more severe psychological challenges, including a higher frequency of SA (*p* = 0.011), although this did not remain statistically significant after adjusting for clinical characteristics related to the ADHD comorbidity [25].

Arias et al*.* found that a comorbid ADHD diagnosis in adults with SUD was significantly associated with SA *(p* < 0.001). This remained significant after correction for multiple comparisons. In a regression analysis, SA was found to be a significant predictor of ADHD (OR 2.25, *p* = 0.0015), with the impact of ADHD being strongest among individuals who had attempted suicide [43].

In a study by Sáez-Francàs et al., CFS patients with persistent ADHD had a significantly higher risk of suicide compared to CFS patients without ADHD (*p* < 0.001) [58].

### Familial and environmental factors

#### Familial risk

##### Self-harm behaviors

In a study conducted by Meza et al*.*, fathers’ negative parenting in childhood significantly correlated with lifetime NSSI [34]. Gordon and Hinshaw investigated if parental distress or dysfunctional parent–child interaction (PSDI) served as significant mediators in the association between ADHD status in childhood and NSSI. They found that parental distress and PSDI did not emerge as mediators of the association between ADHD status and NSSI frequency. PSDI served as a partial mediator in the association between ADHD and NSSI severity, but this did not remain significant after adjusting for externalizing symptoms measured in adolescence [29].

##### Suicide

A large study including 51 707 individuals with ADHD and a matched control cohort (1:5) found that first-degree relatives of probands with ADHD were more likely to have attempted (OR 2.28–2.42) and completed suicide (OR 2.24–2.23) when compared with second-degree and third-degree relatives. The difference in attempted suicide for maternal and paternal half siblings was not statistically significant. After sensitivity analyses, the familial risks were slightly lower, but still statistically significant [50].

Similar parent associated findings were revealed in a Danish longitudinal population study (N = 2.9 million), confirming that parental psychiatric disorders and suicidal behaviors were associated with higher rates of suicidal behavior in their children. If at least one parent had a psychiatric disorder and the child had ADHD, the incidence rate ratios (IRRs) were 7.32 (95% CI [6.49–8.25]), compared to a parent without a registered psychiatric disorder with IRRs of 4.85 (95% CI [4.40–5.35]). The same was found for parental history of suicidal behavior, with IRRs of 3.87 (95% CI [3.9–4.68]) and 2.99 (95% CI [2.75–3.25]), respectively [22].

In the BGALS sample, adverse childhood experiences (ACE) correlated significantly with lifetime SA in females with ADHD [34]. Furthermore, parental distress and PSDI were not found as mediators of the association between ADHD and SA [29]. Having witnessed numerous incidences (≥ 11 times) of parental domestic violence emerged as a significant predictor of SA in a study done by Fuller-Thomson et al*.* [27].

#### Social functioning

Meza et al*.* investigated social preference, peer victimization, and perceived self-competence in relation to SSBs in the BGALS sample [34, 45]. Social preference was found to be negatively associated with SI, SA and NSSI [34, 45]. Perceived self-competence emerged as the sole significant predictor variable for SI *(p* = 0.007) [34]. Self-reported adolescent peer victimization was shown to be a significant partial mediator of the link between RI and NSSI [34, 45]. For SA, perceived self-competence was a dispositional risk factor (*p* = 0.057) [34]. In their study from 2016 [45], teacher-rated adolescent social preference emerged as a partial mediator of the link between RI and SI/SA.

Exploring the Interpersonal Theory of Suicide, Silva et al*.* evaluated ADHD subtypes in connection to thwarted belongingness, perceived burdensomeness, and acquired capability for suicide. Neither adults with predominantly hyperactive/impulsive presentation (ADHD-HI), nor ADHD-I were positively associated with suicide, but rather significantly negatively correlated with thwarted belongingness (*p* < 0.005) and perceived burdensomeness (*p* < 0.05) [48]. ADHD was not significantly associated with acquired capability for suicide [48].

#### Economy and education

Beauchaine et al*.* investigated the impact of financial distress on the association between ADHD and suicide in a Swedish population-based study. Individuals with ADHD who were in the highest default risk bins had a three-fold higher suicide rate than those with ADHD in the lowest default bins [24]. When examining education levels among individuals with ADHD, Fuller-Thomson et al*.* found that those who were post-secondary graduates had 64% lower likelihood of having ever attempted suicide in comparison to those who had not finished high school [27].

## Discussion

Overall, the present review supports the strong association between risk of SSBs and having a diagnosis of ADHD reported in previous reviews and meta-analyses [1–6, 13, 18]. The current study contributes by further investigating factors associated with ADHD that are shown to increase the risk of SSBs.

### Symptom severity

The importance of ADHD-C as a risk factor of SSBs was confirmed by most studies investigating subtypes of ADHD [34, 35, 37, 44, 47, 49]. This could reflect the importance of problems related to the symptom cluster of hyperactivity, inattention, and impulsivity.

However, there is an ongoing discussion regarding distinctions between ADHD subtypes. For instance, Owens et al*.,* state that “*our previous reports revealed extremely few differences by subtype*” [37]. The distinctions are also questioned, based on findings that ADHD subtypes can change over time [59, 60]. Willcutt et al*.* proposed dimensional modifiers reflecting the number of hyperactivity-impulsivity and inattention symptoms at the time of assessment [60]. In the present review, one study found that both inattention- and hyperactivity/impulsivity symptom severity scores were childhood predictors of NSSI and SA [34], supporting the hypothesis that overall ADHD symptom severity are important factors in relation to SSBs.

Considering the unstable nature of ADHD symptomatology and the heterogenous nature of the disorder [9, 37], both the ADHD-C and symptom persistence can be interpreted as indicators of disorder severity. Viewing ADHD in light of symptom severity is also in line with the ICD-11 guidelines, indicating that ADHD symptoms may vary both with chronological age and severity of the disorder.

The expression of ADHD, as well as its development over time, should therefore be considered when determining the risk for SSBs, and this information should be included in prevention strategies from patients’ early age. It should be noted that studies have shown persistent ADHD is associated with severe emotionally impulsive symptoms leading to increased impairments in daily life, and that emotional problems in ADHD tend to increase in adulthood [11, 61].

### ADHD related characteristics

This review finds various aspects of impulsivity, executive functioning and internalizing and externalizing pathology to play a significant role in the risk for SSBs [29, 34, 37, 45, 46, 49]. All these factors can be related to emotional dysregulation, an impairment that has been proposed to be a core component of ADHD [62]. Self-regulatory problems and impulsivity have been central when exploring the link between SSBs and ADHD [2].

A pioneering review on the subject by James et al*.* hypothesized that impulsivity is the driving force of SSBs [18]. The importance of impulsivity is supported by studies showing that childhood ratings of impulsive traits and behaviors are predictors of emotional fluctuations in adulthood [11], and that poor response inhibition is associated with increased risk for SSBs [12, 63].

In the present review, there are some conflicting results. Although one study suggested significant correlations between hyperactivity/impulsivity symptom severity scores measured in childhood and both NSSI and SA in adolescence and adulthood [34], impulsivity was not found to be a significant predictor in another study [20]. The various findings concerning impulsivity can possibly be attributed to different measures, definitions and interpretations of impulsivity as a concept [64, 65].

In a recent review, key findings point to predominant inattention symptoms and internalizing problems in females, while males are more likely to display hyperactive-impulsive symptoms together with externalizing psychopathology [66]. Although the symptom expression of ADHD differs between genders, both presentations can be interpreted as problems related to executive functioning. Additionally, three papers included in the present review found that executive functioning and response inhibition correlate with the prevalence of SSBs, both as mediating and predictive factors [34, 45, 46]. Given executive functions’ role in self-regulatory processes [67], it is thus likely that inclusion of measures of these functions would impact SSBs risk estimates.

Overall, our findings support that dimensions of emotional dysregulation are an important risk factor of SSBs, and that externalizing and internalizing psychopathology can arise as different symptom expressions of ADHD. Emotional dysregulation can increase symptom severity of ADHD, and lead to increased presence of comorbid disorders and problems with activities in daily life [11]. Our findings regarding emotional dysregulation are in accordance with previous research stating that self-harm behavior as a coping mechanism is linked to poor emotion regulation. Self-harm can thus be considered as an avoidance of negative emotions, making suicide as a possible solution [68]. Two previous systematic reviews highlight the association of self-harm leading to later suicide attempts [13, 68], and emotion regulation should therefore be viewed as a central factor in this link.

### Psychiatric comorbidity

This review included a total of 25 articles investigating the role of psychiatric comorbidities. Articles included in the review showed an elevated risk of SSBs in those with comorbid psychiatric disorders [22, 40]. When adjusting for common psychiatric comorbidities, including SUDs, depression, anxiety, BD and personality disorders (PDs), the association between ADHD and SSBs decreased but remained statistically significant in multiple studies [27, 30, 42, 50]. This was partly confirmed by studies comparing groups of a specific psychiatric disorder with and without comorbid ADHD, showing a significant increased risk for anxiety disorders and BD [25, 38, 53–56], while the results are few and conflicting for SUDs and depression [19, 26, 31, 41, 43, 53].

As far as we know, the present review is the first systematic review that thoroughly assesses the role of psychiatric comorbidity in adults and adolescents with ADHD in relation to SSB. With the use of various methods in the included studies, our findings can be considered robust and in line with Septier and Stordeur who hypothesized that psychiatric comorbidities are “*confounding but not responsible”* of the ADHD-SSBs relationship [2]. Regardless of psychiatric comorbidities, individuals with ADHD seem to have an increased risk of SSBs.

The results further indicated that having comorbid externalizing disorders, such as SUDs, as well as internalizing disorders, such as depression and anxiety, are conditions that increase the risk of SSBs in individuals with ADHD. In addition, difficulties in emotional regulation can be viewed as cardinal symptoms in many psychiatric disorders. Various expressions of symptoms and comorbid disorders thus strengthen the hypothesis of emotional dysregulation being a significant potential mediating factor contributing to a higher risk of SSBs in adolescents and adults with ADHD. An important aspect of these disorders is that symptom expression differs between males and females, something that should be taken into consideration in risk estimates of SSBs.

### Gender differences

This review includes six studies investigating gender differences related to SSBs in individuals with ADHD. All studies showed that females have a higher likelihood of SSB than males [22, 23, 27, 32, 36, 50].

Poor social functioning is presented as an important mediating factor between ADHD and SSBs in studies including only females [34, 45]. Kakuzi et al. observed that high self-concept scores on the CAARS are associated with the presence of SI in females, while the association is stronger for impulsivity scores for males [32].

The field of study for both ADHD and SSBs are associated with what can be called a “gender paradox”. It is important to consider that 48% of the total study population of this review are females. The fairly equal gender distribution is a strength of the current review, considering that more males than females are diagnosed with ADHD, at least in childhood [69]. Self-harm is more often reported by females, while more males are shown to commit suicide [27]. Awareness of gender differences is therefore important when assessing the risks of SSBs, especially with regard to symptom expression in individuals with ADHD.

## Familial and environmental factors

Two population studies reported increased risk of suicidal behavior among close family members of individuals with ADHD [50], and that the presence of parental psychiatric disorders and SSBs are associated with higher rates of SSBs in children with ADHD [22]. This supports findings that a family history of suicidal behavior is a risk factor, and that suicidal behavior aggregates in families [70]. In genetic studies, trait-impulsivity has been put forward as a potential explanation [71, 72]. However, environmental factors such as upbringing, ethnic origin, employment status and occupation are also important to consider [71].

Studies included in this review indicate that adverse childhood experiences, including negative parenting, should be considered as significant mediators of the association between ADHD and SSBs [27, 34]. Results from two studies indicated that better social functioning in childhood and adolescence decreases the likelihood of SSBs later in life [34, 45]. On the contrary, poorer social function, such as self-reported peer victimization and lower perceived self-competence [34], as well as financial problems and lower levels of education [24, 27], are suggested to serve as dispositional risk factors for SSBs.

To understand associations between ADHD and SSBs, one should take the bidirectional link between genetic and environmental factors into account. This is in line with the stress-diathesis model, that points to an interaction between environmental stressors and heritable trait susceptibility of SSBs, independent of psychiatric disorders [73]. Both parental history of psychiatric disorders, demographic and environmental factors such as socioeconomic status should therefore be included among predisposing risk factors for SSB in individuals with ADHD.

### Limitations

Several limitations need to be considered before making robust conclusions. First, some of the presented risk factors are supported by only a few studies. Some studies are also limited by small sample sizes, and imbalances regarding gender and geographical origins. Together, these limitations restrict the representativeness of the included findings.

Meta-analyses of the findings would have provided more precise risk estimates. However, we found it challenging to conduct reliable meta-analyses due to the variety of methods used to measure and analyze outcomes, as well as the different terminology regarding SSBs. As more data are generated on the association between ADHD and SSBs, future meta-analyses are called for, especially regarding the relative importance of different psychiatric comorbidities.

Considering that ADHD symptom expression seem to be related to a wide range of familial and environmental factors, further studies on risk factors associated with SSBs and ADHD should consider inclusion of symptoms at a dimensional rather than diagnostic level. The factors found in this review are also likely to interact with each other, making it challenging to establish independent effects. Due to symptom overlap, it may also be challenging to differentiate ADHD from disorders such as BD and PDs. Since all papers specifically assessing drug efficiency or treatment were excluded, medication use could be a potential confounding or mediating factor that has not been accounted for in this review.

## Conclusion

This is the first systematic PRISMA review investigating a broad range of risk factors triggering SSBs in adolescents and adults with a clinical diagnosis of ADHD. By this, the review expands on findings reported in previous reviews.

Factors found to increase the risk of SSBs include ADHD symptom severity and persistence, gender, family history of ADHD, childhood and parental influences, and social functioning. When adjusting for psychiatric comorbidities, adolescents and adults with ADHD still had an increased risk of SSBs.

Overall, ADHD emerges as an independent risk factor for SSBs. Awareness of psychiatric comorbidities, symptom expression, and other risk factors associated to SSBs should have clinical implications in terms of screening and suicide prevention strategies in adolescents and adults with ADHD. Future longitudinal studies investigating the relative strengths of the risk factors reported in the present study and the impact of preventive strategies on the life of individuals along the full spectrum of ADHD symptom severity are called for.

### Footnotes

The review protocol was not registered prior to conducting the literature search.

### Supplementary Information

**Additional file 1.** 

## Data Availability

All data analysed in this systematic review are included in this published article [and its supplementary information files].

## Abbreviations

AAQoL: Adult ADHD Quality of Life Questionnaire
ACE: Adverse childhood experiences
ADHD: Attention deficit/hyperactivity disorder
ADHD-C: ADHD-combined
ADHD-HI: ADHD-Hyperactivity/impulsivity
ADHD-I: ADHD-inattention
APD: Antisocial personality disorder
ASD: Autism spectrum disorder
ASRS: Adult ADHD Self-Report Scale
BD: Bipolar disorder
BGALS: Berkeley Girls with ADHD Longitudinal Study
BPD: Borderline personality disorder
CAADID: Conners’ Adult ADHD Diagnostic Interview for DSM-IV
CAARS: Conners’ Adult ADHD Rating Scales
CBCL: Child behavior checklist
CFS: Chronic fatigue syndrome
CI: Confidence interval
CS: Completed suicide
DIVA: Diagnostic Interview for ADHD in Adults
DSH: Deliberate self-harm
DSM: Diagnostic and Statistical Manual of Mental Disorders
EF: Executive function
EUPD: Emotionally unstable personality disorder
GAD: General anxiety disorder
HDRS: Hamilton Depression Rating Scale
HR: Hazard ratio
ICD: International classification of diseases
ID: Intellectual disability
IRR: Incidence rate ratio
K-SADS-PL: Kiddie- Schedule for Affective Disorders and Schizophrenia present-life version
MADRS-S: Montgomery-Asberg Depression Rating Scale–Self
MDD: Major depressive disorder
MINI: Mini-International Neuropsychiatric Interview
NS: Nonsignificant
NSSI: Non-suicidal self-harm
OCD: Obsessive–compulsive disorder
OR: Odds ratio
*p*: Probability value
PaD: Panic Disorder
PD: Personality disorder
PTSD: Post-traumatic stress disorder
PSDI: Dysfunctional parent–child interactions
RI: Response inhibition
SA: Suicide attempt
SAD: Social anxiety disorder
SCID: Structured Clinical Interview for DSM
SE: Standard error
SI: Suicidal ideation
SIB: Self-injurious behavior
SNAP: Swanson, Nolan, and Pelham Questionnaire
SSADDA: Semi-structured Assessment for Drug Dependence and Alcoholism
SSBs: Suicidal spectrum behaviors
SUAS: Suicide assessment scale
SUD: Substance use disorder
TS: Tourettes syndrome
WURS: Wender Utah Rating Scale
SNAP: Swanson, Nolan, and Pelham Questionnaire

## Declarations

**Ethical approval and consent to participate**

Not applicable.
